# Supplementary material for: Effectiveness and Safety of Ayurvedic Medicines in Type 2 Diabetes Mellitus Management: A Systematic Review and Meta-Analysis
Source: Front Pharmacol. 2022 Jun 8;13:821810. doi: 10.3389/fphar.2022.821810 (PMC9213670; doi:10.3389/fphar.2022.821810)
Supplement: Supplementary file 1 [file DataSheet1.zip › Supplementary Material/Forest and Funnel Plots/Syzygium cumini/Syzygium cumini.pdf]

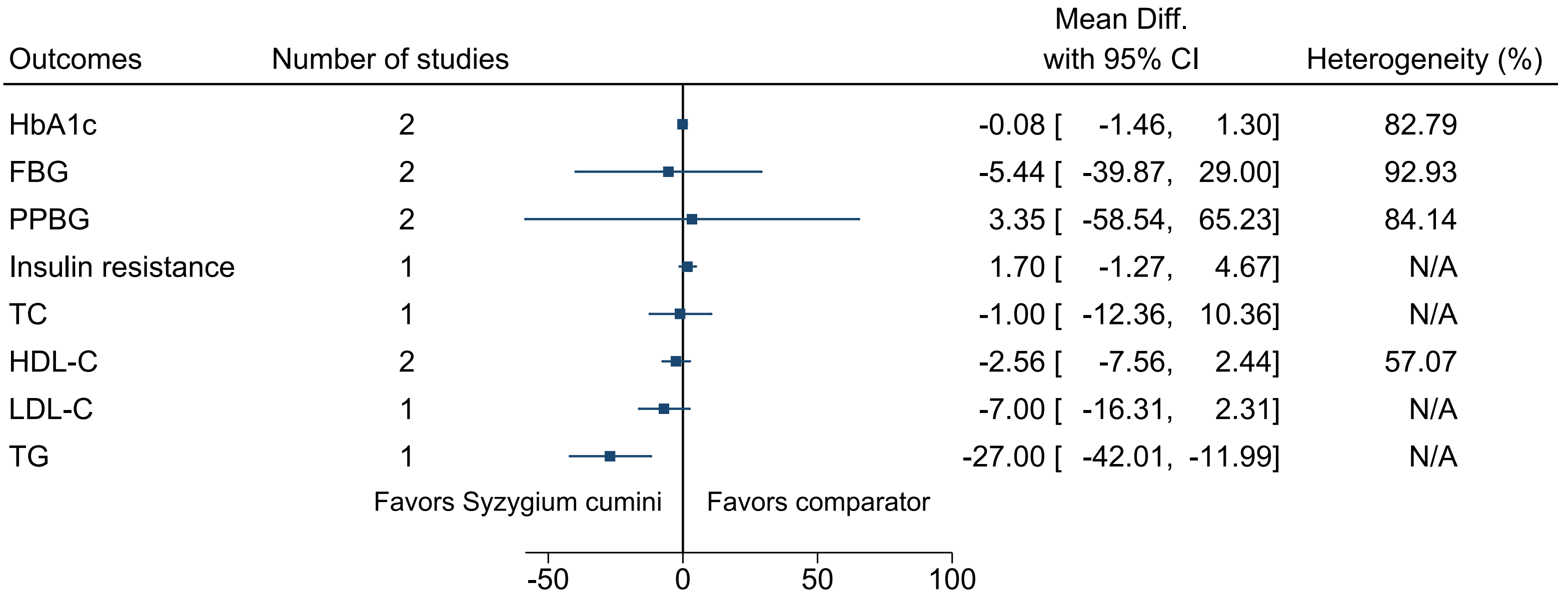
Outcomes
Number of studies
Mean Diff.
with 95% CI
Heterogeneity (%)
HbA1c 2 -0.08 [-1.46, 1.30] 82.79
FBG 2 -5.44 [-39.87, 29.00] 92.93
PPBG 2 3.35 [-58.54, 65.23] 84.14
Insulin resistance 1 1.70 [-1.27, 4.67] N/A
TC 1 -1.00 [-12.36, 10.36] N/A
HDL-C 2 -2.56 [-7.56, 2.44] 57.07
LDL-C 1 -7.00 [-16.31, 2.31] N/A
TG 1 -27.00 [-42.01, -11.99] N/A
Favors Syzygium cumini
Favors comparator
-50
0
50
100
